# Evaluation of the local tolerance and systemic safety of a novel intravaginal probiotic product in cows

**DOI:** 10.1007/s11259-026-11264-7

**Published:** 2026-05-11

**Authors:** György Csikó, Orsolya Palócz, Zsóka Várhidi, Péter Sátorhelyi, Balázs Erdélyi, Viktor Jurkovich

**Affiliations:** 1 Department of Pharmacology and Toxicology, University of Veterinary Medicine, Budapest, Hungary; 2 Department of Animal Hygiene, Herd Health and Mobile Clinic, University of Veterinary Medicine, Budapest, Hungary; 3 Fermentia Microbiological Research and Development Ltd, Budapest, Hungary; 4 Centre for Animal Welfare, University of Veterinary Medicine, Budapest, Hungary

**Keywords:** Intravaginal probiotics, Cow, Safety, Local tolerance

## Abstract

Since its discovery, the microbiota has been increasingly recognised for its role in maintaining health and contributing to various disease conditions. In the reproductive tract, microbial populations can significantly influence endometrial health, internal homeostasis, and fertility. The preservation or restoration of a balanced microbiota through appropriate probiotic products may support reproductive health; in addition, candidate probiotics must be demonstrated to be safe for use. The aim of this study was to assess the local tolerance and systemic safety of a novel intravaginal probiotic product in cows. Twenty-four animals were enrolled and assigned to four groups: single-dose, three-, and five-fold dose of the test product, and a placebo group receiving excipients only. Physical examination and evaluation of the vaginal mucosa were conducted prior to each treatment, one day after and one and two weeks after the final probiotic administration. Blood and urine samples were collected before treatment and following treatment: one day after the last administration of intravaginal probiotic and again two weeks post-treatment. Blood haptoglobin, serum amyloid A, glucose, non-esterified fatty acids, and beta-hydroxybutyrate were measured to assess inflammatory and metabolic responses, and differences over time and between groups were statistically analysed. No dose-dependent systemic changes were observed; however, transient, time-related alterations were noted across all groups, including controls. The investigational probiotic product was well tolerated both locally and systemically, with tolerability comparable across all groups, including the placebo. These data establish a safety profile in healthy cows and support further investigation of this product in studies focusing on efficacy and microbiome modulation.

**Supplementary Information:**

The online version contains supplementary material available at 10.1007/s11259-026-11264-7.

## Introduction

Microbiome research has advanced rapidly over recent decades, becoming a central topic in both science and public health. Microbiomes are defined as communities of commensal, symbiotic, and pathogenic microorganisms that inhabit specific body niches. In addition to the microbial organisms themselves, the microbiome encompasses associated structural components, metabolites, and their interactions with the surrounding environmental conditions (Berg et al. 2020). The term *microbiota* refers specifically to the living microbial members of these ecosystems, which vary according to anatomical and physiological characteristics.

Probiotics—live microorganisms conferring health benefits to the host—play a key role in maintaining microbial homeostasis by colonising tissues and inhibiting pathogen proliferation (Hou et al. 2022). Their beneficial effects extend beyond the gastrointestinal tract; certain strains have shown positive impacts on genital health (Várhidi et al. 2024). Most postpartum uterus diseases begin with the bacterial contamination of the uterine lumen (Sheldon et al. 2006). The administration of intravaginal probiotic formulation might decrease the growth of pathogenic bacteria. In veterinary medicine, probiotic supplementation has demonstrated efficacy in reducing postpartum uterine disorders in cows and buffalos (Gohil et al. 2023; Madureira et al. 2023).

Mounting evidence supports the use of probiotics to promote health, inhibit pathogenic growth, and enhance productivity in animals (Rosales and Ametaj 2021; Adnane et al. 2024; Hashem and Gonzalez-Bulnes 2022). The increasing demand for safe, naturally derived alternatives to antibiotics has further driven their development (Kwoji et al. 2021). Conventional antibiotic treatments for endometritis show inconsistent success and are limited by withdrawal periods, costs, and the growing threat of antimicrobial resistance (Várhidi et al. 2024; Adnane et al. 2024). These challenges underscore the need for effective non-antibiotic therapies (Sarkar et al. 2016).

**Fig. 1:**
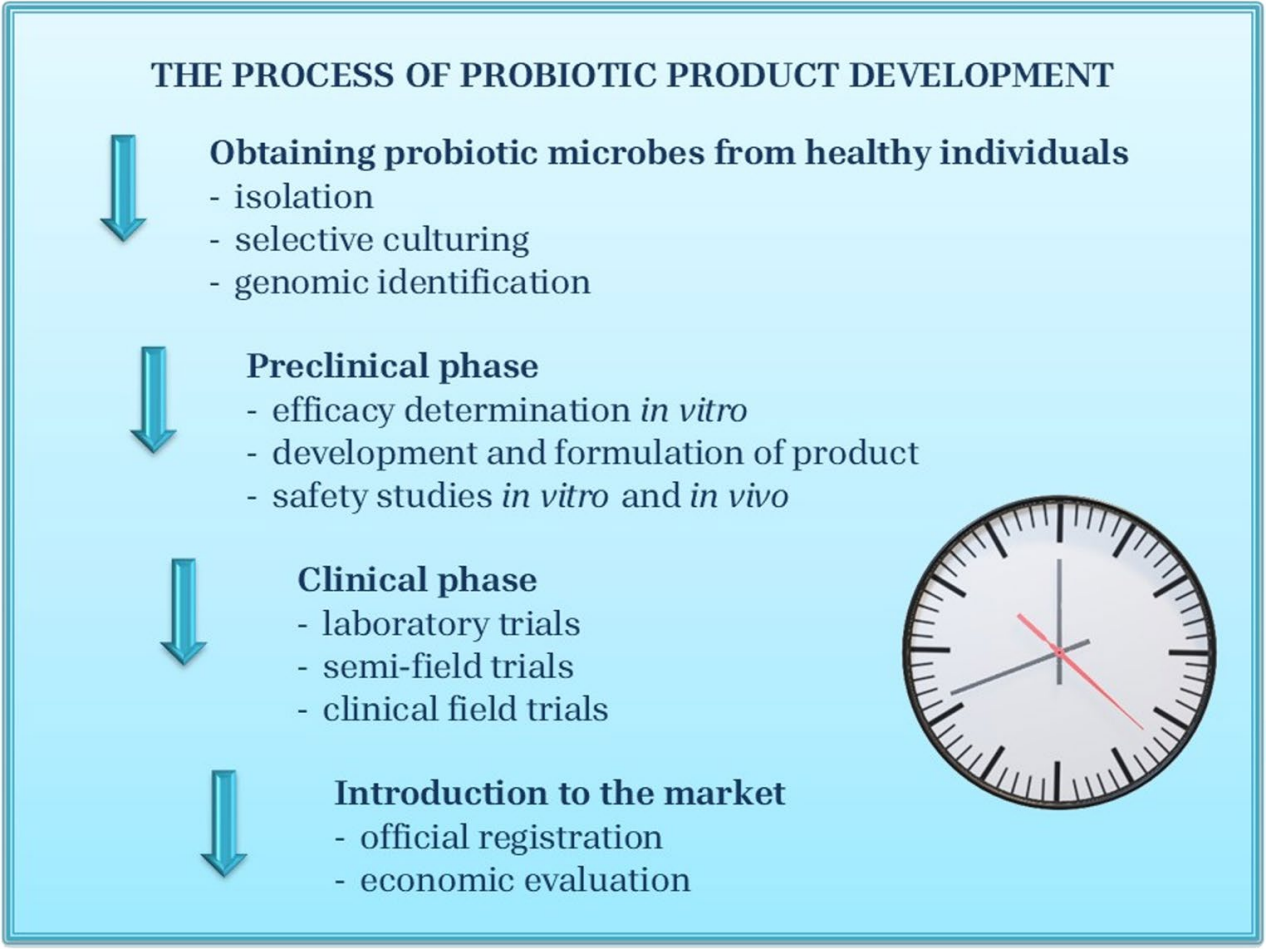
Main steps of the research and development of a new probiotic product. (created according to Cunningham et al. 2021 and Zuntar et al. 2020)

Probiotics are increasingly regarded as “living drugs” with potential to reduce antibiotic dependence. However, uncertainties regarding optimal dosage, timing, and safety profiles continue to hinder their widespread clinical adoption. During the development and regulatory approval process of novel probiotics, it is insufficient to demonstrate efficacy alone (Fig. 1). As antibiotic use becomes more restricted, ensuring that novel probiotic formulations are both effective and safe remains a critical step toward their integration into veterinary practice (Merenstein et al. 2023).

Prior to market authorization, European veterinary medicinal legislation mandates that all veterinary medicinal products demonstrate both safety and efficacy for their intended use in food-producing or companion animals (Jukes 2011; Elliot 2024). In the case of microbe-based preparations used in therapy, it is also a basic requirement that they do not have an adverse effect on the treated animals. Although generally regarded as safe, probiotics may theoretically induce a range of side effects, including systemic infections (Doron and Snydman 2015), harmful metabolic activities, excessive immune activation in susceptible individuals, disruption of the host’s resident microbiota and horizontal gene transfer (Kothari et al. 2019). Furthermore, specific probiotic strains may harbour undesirable characteristics such as virulence factors, e.g. haemolytic activity and the capacity to produce toxic metabolites (Rossi et al. 2019), or transferable antimicrobial resistance genes (Mathur and Singh 2005). In rare cases, probiotics may translocate into the systemic circulation, potentially leading to invasive infections (Alayande et al. 2020; Merenstein et al. 2023). Additionally, the use of some probiotic formulations theoretically can induce local irritation at the site of application (Czaja et al. 2007). Therefore, it is essential to demonstrate not only systemic safety but also local tolerability. An ideal probiotic should exhibit several key characteristics, including non-pathogenicity, non-toxicity, and demonstrable benefits to the host organism. Importantly, even when administered at high concentrations, a microbial strain lacking overt virulence traits must not exert any detrimental effects on host health.

The aim of our current study was to investigate the local tolerance and systemic safety of a newly developed intravaginal probiotic preparation intended for the control of postpartum metritis in cows. The intended application of the product is to maintain the health of reproductive tract and support microbial homeostasis, potentially contributing to the prevention and treatment of postpartum uterine disease. The strains included in this product (*Limosilactobacillus fermentum*,* Lactiplantibacillus plantarum*,* Limosilactobacillus reuteri*,* Lacticaseibacillus rhamnosus*,* Pediococcus pentosaceus*, and *Brevibacillus parabrevis*) were selected based on prior in vitro screening for safety and compatibility with the bovine reproductive tract. The included lactic acid bacteria contribute to the maintenance of a low vaginal pH through lactic acid production, inhibiting opportunistic pathogens such as *Escherichia coli*, *Trueperella pyogenes*, and *Staphylococcus aureus* (Adnane and Chapwanya 2022). Several strains (*L. fermentum*, *L. reuteri*, *L. plantarum*, and *P. pentosaceus*) are known to produce bacteriocins and other antimicrobial compounds (e.g., reuterin, pediocins), enhancing their protective efficacy (Vinayamohan et al. 2024). *L. rhamnosus* and *L. reuteri* exhibit strong adhesive and immunomodulatory capacities, promoting epithelial barrier integrity and competitive exclusion of pathogens (Tukaram et al. 2025). *Brevibacillus parabrevis* was included in the preparation because it had previously been identified as a commensal component of the bovine vaginal microbiome and exhibited no antimicrobial resistance to antibiotics commonly used in cattle (Várhidi et al. 2025). Dosages were chosen according to the projected therapeutic dose (1×), with 3× and 5× supratherapeutic doses included to provide safety margins.

The specific objective of this study was to evaluate systemic biochemical and haematological parameters, as well as local tolerance, in cows receiving intravaginal probiotics at different dose levels, in line with the European Medicines Agency (EMA) and VICH guidelines on target animal safety. While efficacy, microbiome modulation, or cellular mechanisms were beyond the scope of this first safety evaluation, these aspects are planned for subsequent investigations.

## Materials and methods

### Examination of animals

In total, 24 healthy, non-pregnant Holstein Friesian dairy cows selected for culling were enrolled in the study, cows with similar parity, DIM, and average milk yield were randomly assigned to 4 groups on the week before enrolment (Table 1), on a Hungarian large-scale dairy farm. The animals were at metoestrus or dioestrus stage at the time of treatment. In the design and implementation of the experiment, we adhered to the recommendations and methodological guidelines established by the European Medicines Agency, which specify the required animal numbers, groupings, and parameters to be evaluated according to VICH GL9 (2000) and GL43 (2009) and EMA (2016). Guideline on non-clinical local tolerance testing of medicinal products. Physical examination, including rectal temperature measurement, vaginoscopy, scoring vaginal discharge (VD) on a scale of 0–3 (Sheldon et al. 2006, 2009), body condition scoring on a scale of 1–5 (Ferguson et al. 1994) and locomotion scoring on a scale of 1–5 (Sprecher et al. 1997) was completed as a first step to ensure that the selected animals were healthy (Table 1). Cows were matched according to their data to create homogeneous groups (Table 1). Then the cows were randomly assigned to four groups: control (excipients only, *n* = 6); 1× (the intended dose, *n* = 6); 3× (three-fold of the intended dose, *n* = 6); 5× (five-fold of the intended dose, *n* = 6).

**Table 1:** Data of the animals enrolled in the study (mean ± SD)

Group	Control	1×	3×	5×
Parity	2.0 ± 0.9	1.8 ± 0.8	1.8 ± 1.3	2.2 ± 1.9
DIM	467.7 ± 129.8	456.5 ± 69.6	441.2 ± 102.4	482.0 ± 96.8
Last milk (L/day)	26.6 ± 10.6	24.3 ± 6.5	23.2 ± 4.5	25.9 ± 6.5
BCS	3.1 ± 0.4	3.6 ± 0.7	3.2 ± 0.5	3.5 ± 1.0
Locomotion score	2.3 ± 0.5	2.0 ± 0.9	2.2 ± 1.0	2.0 ± 0.9

DIM: days in milk; last milk: average milk yield during the week before the enrolment; BCS: body condition score

Treatment of animals

**Fig. 2:**
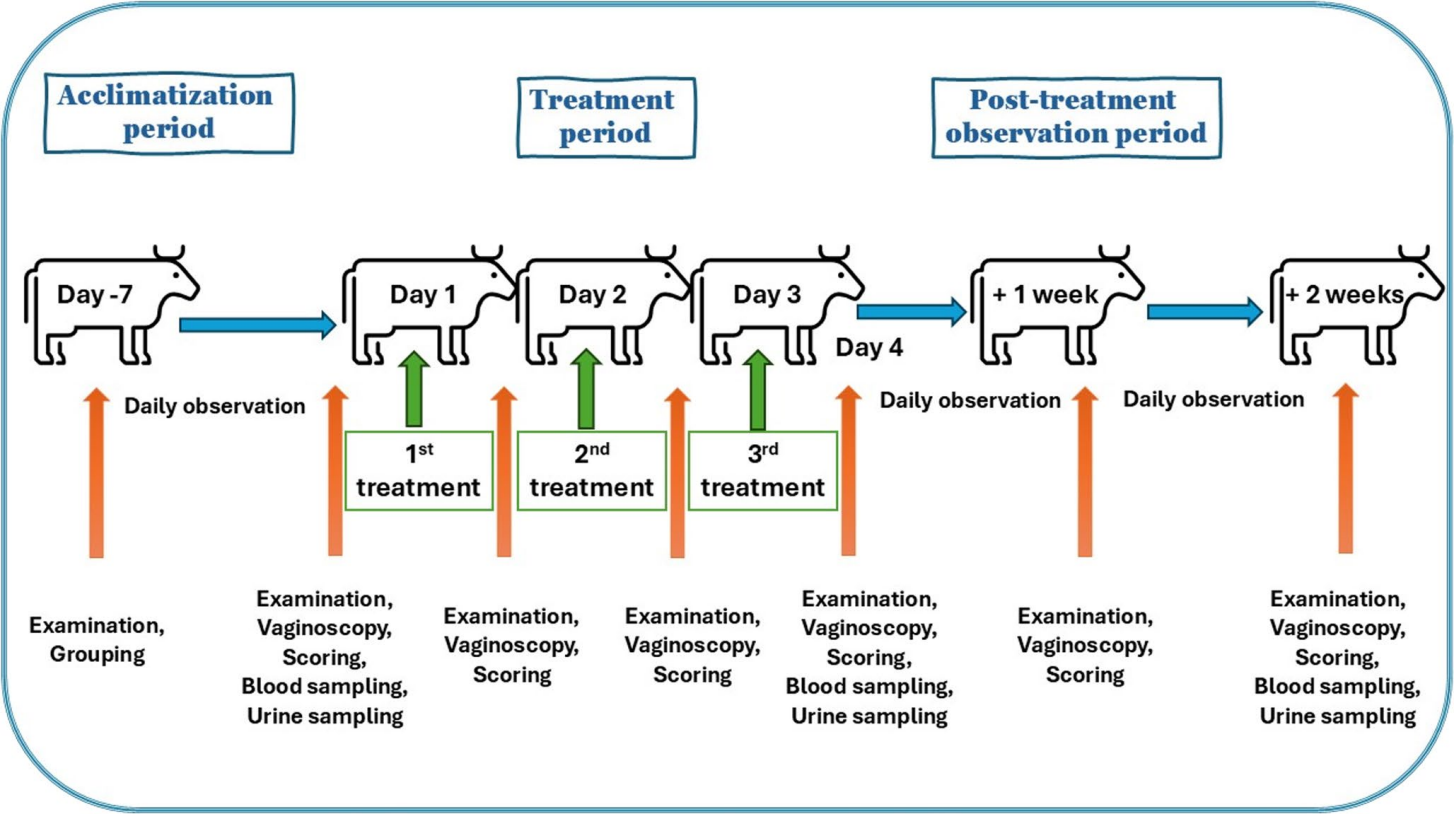
Experimental design for the assessment of tolerance of intravaginal probiotic treatment in dairy cows

The investigational test product (Table 2) was injected into the vagina near the cervix of the examined animals from sterile syringes, using sterile plastic sheaths for artificial insemination (Zhejiang Kangrui Apparatus Technology Co., Ltd., Zheijang, China), every day once, for three consecutive days, as long as the planned therapeutic dosage. For preventing contamination during the application, the cow’ vulva was gently cleaned with water, then iodine (Betadine solution, EGIS Ltd, Budapest, Hungary), then 70% alcohol. During the treatments, 10 mL of the preparation was administered in each case, and the concentration of viable bacteria was increased proportionally in the 3× and 5× groups. We performed the following measurements and samplings during the study. Body condition scoring (Ferguson et al. 1994), rectal temperature measurement (VT 1831 Digital veterinary thermometer, Microlife A.G., Widnau, Switzerland), visual examination of the vaginal mucosa through a speculum were performed before all product administrations, and one and two weeks after the last administration. Blood and urine samples were taken before the first administration, one day after the last administration and two weeks after the last administration of treatment (Fig. 2).

Blood samples were collected from the coccygeal vein into EDTA-, sodium fluoride (NaF)-containing and additive-free (plain) blood collection tubes (Vacuette; Greiner Bio-One International GmbH, Kremsmünster, Austria). Haematological parameters were measured using an automated haematology analyser (Sysmex XN-1500 V; Sysmex Corporation, Kobe, Japan). Serum biochemical analyses (Table S1) were performed using an automated clinical chemistry analyser (Advia 1800; Siemens Healthcare Diagnostics, Tarrytown, NY, USA). The measured parameters were; glucose, aspartate aminotransferase (AST), alanine aminotransferase (ALT), alkaline phosphatase (ALKP), gamma glutamyl transferase (GGT), total bilirubin, direct bilirubin, total protein, albumin, albumin/globulin ratio, serum amyloid A, haptoglobin, total cholesterol, triglyceride, beta hydroxybutyrate (BHB), non-esterified fatty acids (NEFA), urea, creatinine, inorganic phosphate, sodium, potassium, calcium, magnesium, chloride, iron, creatine kinase (CK), and lactate dehydrogenase (LDH).

Urine was collected opportunistically at the time of blood sampling, when cows were naturally urinating. Mid-stream samples were obtained whenever possible, using sterile tubes (V-Monovette, Sarstedt AG & Co. KG, Nümbrecht, Germany). The urine samples were tested with a test strip intended for veterinary use (Kruusevet-10 Urine Strips, Lot. 220511, Jorgen Kruuse A6S, Langeskov, Denmark) for measuring urobilinogen, glucose, bilirubin, ketone, density, blood, pH, protein, nitrate, and white blood cells.

**Table 2:** Composition of the intended dose (1×) of the investigational test product

Excipients	1.5% HEC, 1.0% dextrose in PBS buffer; pH = 6.0
Prebiotic component	1.0% Inulin
Probiotic components	*Limosilactobacillus fermentum* 1 × 10^8^ CFU/dose*, *Lactiplantibacillus plantarum* 1 × 10^8^ CFU/dose, *Limosilactobacillus reuteri* 1 × 10^8^ CFU/dose, *Lacticaseibacillus rhamnosus* 1 × 10^8^ CFU/dose, *Pediococcus pentosaceus* 1 × 10^8^ CFU/dose, *Brevibacillus parabrevis* 5 × 10^8^ CFU/dose

HEC – hydroxyethyl cellulose, PBS – phosphate buffered saline, CFU – colony forming unit

*Volume of the dose was always 10 mL. In case of the 3× and 5× doses proportionally higher CFUs were applied in the same volume of the excipients, and the control animals received the excipients only

A total of 24 healthy dairy cows (462 ± 99 DIM) divided into 4 groups (i.e., Control, 1×, 3×, and 5×; *n* = 6/group). A total of 10 ml product was administered intravaginally once daily for three consecutive days (days 1–3). The 1× group received the projected therapeutic dose of viable bacteria, while the 3× and 5× groups received supratherapeutic doses to assess safety margins, control animals received the excipients only. Blood and urine sampling were performed at three time points: baseline (day 0), one day after the last administration (day 4), and two weeks after the last administration (day 17). On treatment days, the examinations were performed immediately before administration of the test product.

### Statistical analysis

Statistical analyses were performed using R version 4.4.3 (R Core Team, 2025). The study design involved four treatment groups with repeated measurements across three time points (before treatment, one day after the last administration, and two weeks after).

The distribution of continuous variables was assessed using the Shapiro–Wilk test. Variables with *p* > 0.05 were considered normally distributed, whereas those with *p* ≤ 0.05 were regarded as non-normally distributed.

The following parameters showed a normal distribution: rectal temperature, ALT (alanine aminotransferase), ALKP (alkaline phosphatase), GGT (γ-glutamyl transferase), total cholesterol, triglycerides, sodium, magnesium, chloride, iron, white blood cell count (WBC), lymphocyte count, red blood cell count (RBC), haemoglobin (HGB), haematocrit (HCT), and platelet count (PLT).

Parameters with a non-normal distribution included AST (aspartate aminotransferase), total protein, albumin, albumin/globulin ratio, serum amyloid A, haptoglobin, glucose, β-hydroxybutyrate (BHB), non-esterified fatty acids (NEFA), urea, creatinine, phosphorus, potassium, calcium, creatine kinase (CK), lactate dehydrogenase (LDH), neutrophil, monocyte, eosinophil, and basophil counts, as well as urine specific gravity and urine pH.

For normally distributed variables, differences over time (repeated measures) and between treatment groups were analysed using two-way repeated measures ANOVA, followed by Tukey’s post hoc test where applicable. For non-normally distributed variables, the Friedman test was applied to assess changes over time, and the Kruskal–Wallis test was used for between-group comparisons; in both cases followed by Dunn’s multiple comparison test. Data are expressed as mean ± standard deviation (SD) for normally distributed variables, and as median (interquartile range) for non-normally distributed variables. Statistical significance was considered at *p* < 0.05.

Sample size was determined in accordance with EMA and VICH guidelines for target animal safety studies, which prescribe minimum numbers per group to ensure adequate safety assessment. Although not powered for efficacy outcomes, the study design was considered sufficient for detecting treatment-related adverse effects.

## Results

No treatment-related clinical signs were observed. Rectal temperature, body condition score, locomotion score, and vaginal mucosa appearance remained within normal limits across all groups throughout the study. Vaginal discharge was not observed in any of the animals at any sampling time. Haematological values—including white blood cell (WBC) count, differential leukocyte counts (neutrophils, lymphocytes, monocytes, eosinophils, basophils), red blood cell (RBC) count, haemoglobin concentration, haematocrit, and platelet count—remained within physiological ranges throughout the study. Urinalysis showed no clinical abnormalities; all samples were negative for urobilinogen, bilirubin, glucose, ketones, and white blood cells at all sampling times.

Descriptive biochemical, haematological, and urine pH values at each time point are presented in Tables S2–S4. Longitudinal statistical analysis indicated that several parameters exhibited significant time effects, whereas no consistent group effects or time × group interactions were detected. Therefore, data from all groups were pooled to illustrate the overall effect of time (Table 3).

Alanine aminotransferase (ALT) showed a significant time effect (*p* < 0.0001), increasing one day after the last administration across all groups and returning toward baseline by two weeks. Urea concentrations likewise demonstrated a significant time effect (*p* < 0.0001), with decreased values observed at post-treatment sampling times across groups, including controls. Changes over time were also detected for selected metabolic markers, including beta-hydroxybutyrate (BHB), non-esterified fatty acids (NEFA), and triglycerides, as well as for creatinine (*p* < 0.05). However, no interaction was observed between the group and time. A single low lactate dehydrogenase (LDH) outlier was observed in one cow without associated clinical signs.

Rectal temperature showed minor fluctuations over time, with statistically significant time effects detected; however, no group-specific or interaction effects were identified. Albumin and glucose concentrations also exhibited significant time-related changes one day after treatment across groups (albumin: *p* < 0.05; glucose: *p* < 0.05). Electrolyte concentrations showed limited variability; sodium, potassium, chloride, and iron concentrations exhibited statistically significant time effects in longitudinal analysis, without treatment group or time × group interaction effects.

Haematological parameters showed several transient changes, including WBC, lymphocyte, monocyte, and haemoglobin values, while remaining within physiological ranges. Urine specific gravity showed a significant time effect, primarily driven by changes observed in control animals.

**Table 3:** Pooled time-course changes in rectal temperature, clinical chemistry, haematology, and urinalysis parameters across all cows

Parameter	Unit	Reference range	Before	After	Two weeks after	Time effect (*p*)
Rectal temperature	°C	—	38.05 ± 0.33^a^	38.49 ± 0.27^b^	38.37 ± 0.38^b^	< 0.05
Alanine Aminotransferase (ALT)	U/L	10–35	34.67 ± 7.86^a^	40.04 ± 8.15^b^	37.96 ± 6.29^a^	< 0.0001
Albumin	g/L	25–38	32.45 (30.85–33.32)^a^	33.35 (31.88–34.12)^b^	32.35 (30.65–33.32)^a^	< 0.05
Glucose	mmol/L	2.2–5.5	4.35 (4.1–4.5)^a^	4.8 (4.6–4.8)^b^	4.55 (4.4–4.7)^ab^	< 0.05
Triglycerides	mmol/L	0.08–0.20	0.06 ± 0.025^a^	0.16 ± 0.036^b^	0.15 ± 0.031^b^	< 0.001
Beta-Hydroxybutyrate (BHB)	mmol/L	< 1.0	0.69 (0.62–0.81)^a^	0.34 (0.29–0.41)^b^	0.42 (0.39–0.53)^c^	< 0.001
Non-Esterified Fatty Acids (NEFA)	mmol/L	< 0.4	0.095 (0.08–0.11)^a^	0.215 (0.16–0.37)^b^	0.17 (0.12–0.24)^a^	< 0.05
Urea	mmol/L	3–8	6.55 (6.3–6.9)^a^	3.15 (2.8–3.3)^b^	4.0 (3.7–4.2)^c^	< 0.0001
Creatinine	µmol/L	40–148	90 (85.8–95.5)^a^	86 (78.8–89)^b^	90 (88.75–93.3)^a^	< 0.05
Sodium	mmol/L	132–152	141.88 ± 1.18^a^	140.5 ± 1.21^b^	140.45 ± 1.92^b^	< 0.05
Potassium	mmol/L	3.9–5.8	4.6 (4.5–4.8)^a^	4.9 (4.7–5.1)^b^	4.7 (4.6–4.9)^a^	< 0.05
Chloride	mmol/L	95–110	103.46 ± 2.06^a^	105.58 ± 1.5^b^	102.41 ± 2.16^a^	< 0.001
Iron	µmol/L	20–35	24.18 ± 5.27^a^	23.17 ± 3.59^a^	28.24 ± 3.35^b^	< 0.01
White blood cells (WBC)	cells/L	4.0–12.0 × 10^9^	9.5 ± 2.36^a^	8.27 ± 1.9^b^	8.29 ± 2.09^b^	< 0.05
Lymphocytes	cells/L	2.0–7.0 × 10^9^	3.99 ± 1.23^a^	3.48 ± 1.03^b^	3.68 ± 1.23^b^	< 0.01
Monocytes	cells/L	0.025–0.84 × 10^9^	0.45 (0.4–0.53)^a^	0.3 (0.28–0.42)^b^	0.4 (0.4–0.5)^a^	< 0.01
Haemoglobin	g/L	80–150	107.38 ± 7.13^a^	112.17 ± 9.69^b^	109.42 ± 7.92^a^	< 0.05
Urine specific gravity		1.02–1.045	1.02 (1.02–1.03)^a^	1.02 (1.01–1.02)^b^	1.02 (1.01–1.02)^b^	< 0.05

Data are presented as mean ± SD for normally distributed variables and median (interquartile range) for non-normally distributed variables. Different superscript letters within a row indicate statistically significant differences between time points based on pairwise post hoc comparisons (Tukey’s post hoc test following repeated-measures ANOVA or Dunn’s multiple comparison test following Friedman analysis, as appropriate). The overall effect of time is shown in the final column. Reference ranges are as reported by the analytical laboratory

## Discussion

Although probiotics are generally recognised as safe, their efficacy and safety are strain specific, and safety cannot be assumed for newly developed formulations without appropriate evaluation. Therefore, the present study assessed both local tolerance and systemic safety of a novel intravaginal probiotic product in cows, including supratherapeutic dose levels, in accordance with EMA and VICH target animal safety guidelines.

In this study, no treatment-specific local or systemic adverse effects were observed. Importantly, this does not imply that no changes occurred in measured parameters; rather, the observed biochemical and haematological changes were mild, transient, and non–dose-dependent, and were not associated with clinical signs or pathological findings. All animals remained clinically healthy throughout the study, and local tolerance was confirmed by the absence of vaginal irritation or discharge in all groups. These findings are consistent with prior studies evaluating the safety of intravaginal probiotics in animal models. For instance, Silva et al. (2023) reported the absence of inflammatory responses and only minor ultrastructural changes in mice administered *Lacticaseibacillus rhamnosus* CRL1332. No instances of vaginal discharge were observed during our study, and previous research has shown that intravaginal administration of *Lactobacillus sakei* and *Pediococcus acidilactici* reduced the incidence of foul-smelling vaginal discharge in dairy cows three weeks postpartum (Ametaj et al. 2014). Similarly, Gohil et al. (2023) and Miranda et al. (2021, 2024) demonstrated the absence of adverse reactions with probiotic use in cows; however, their studies did not include supratherapeutic dose groups or extensive systemic laboratory testing.

Several biochemical and haematological variables exhibited statistically significant time-related changes, as demonstrated by longitudinal analysis. These changes occurred similarly in control and treated animals and were not accompanied by significant group or time × group interaction effects, indicating that they were not attributable to probiotic dose, suggesting that observed changes were not dose-dependent. In this context, the term mild refers to changes that remained within, or only marginally deviated from, established physiological reference ranges and resolved spontaneously during the observation period. Such transient fluctuations are commonly observed in longitudinal safety studies and may reflect procedural stress, repeated handling, sampling-related effects, or short-term metabolic adaptation rather than treatment-induced toxicity (Siska et al. 2017).

For example, alanine aminotransferase (ALT) increased transiently one day after the final administration in all groups, including controls, and returned toward baseline within two weeks. The absence of concurrent changes in other liver-associated enzymes (AST, GGT, ALKP) and the lack of clinical signs strongly suggest that this finding was not indicative of hepatocellular injury. Similarly, urea, beta-hydroxybutyrate (BHB), non-esterified fatty acids (NEFA), triglycerides, creatinine, and selected electrolytes exhibited time-dependent changes without dose-related patterns, supporting the interpretation of physiological rather than toxicological relevance.

Transient alterations in metabolic markers such as BHB and NEFA have been reported previously in dairy cows under varying physiological or management-related conditions, including handling, dietary variation, or stress associated with repeated sampling (Sundrum 2015; Rosales and Ametaj 2021). In the present study, these parameters remained within acceptable physiological limits and showed no evidence of cumulative or progressive deviation with increasing probiotic dose. Likewise, modest time-related fluctuations in sodium, potassium, chloride, and iron concentrations were observed without any consistent group effect, suggesting normal homeostatic regulation rather than treatment-related disturbance.

Haematological parameters also showed some transient time-related variation; however, all values remained within physiological ranges. Changes in WBC counts or differential leukocyte populations were not dose dependent and were not accompanied by clinical signs of inflammation or infection. Acute phase proteins, including serum amyloid A and haptoglobin, remained low throughout the study, further supporting the absence of systemic inflammatory responses. Due to the complete absence of characteristic clinical symptoms and partly for animal welfare reasons, in this study, histopathological examinations (Siska et al. 2017) were not performed.

Overall, the lack of dose-dependent effects, absence of time × group interactions, and consistency of changes across control and treated animals provide strong evidence that the investigational probiotic product did not induce clinically relevant local or systemic adverse effects, even at three- and five-fold supratherapeutic doses. These findings are consistent with previous studies reporting good tolerability of intravaginal probiotic preparations in cattle (Ametaj et al. 2014; Miranda et al. 2021; Gohil et al. 2023), while extending the available safety data by including higher dose levels and comprehensive systemic laboratory assessment. Cows included in the study were in metoestrus or dioestrus at the time of treatment. Although these stages differ in progesterone concentrations, vaginal pH in cattle remains relatively stable throughout the oestrous cycle (Schilling and Zust 1967) and is therefore unlikely to substantially influence vaginal microbiota composition or local tolerance in clinically healthy animals. Accordingly, the stage of the oestrous cycle was not expected to affect the safety outcomes of the present study.

Some limitations of the present study should be acknowledged. The study was designed in accordance with EMA and VICH guidelines for target animal safety evaluation and was therefore not powered to detect efficacy-related outcomes. Only clinically healthy cows were included, and microbiome composition or clinical efficacy endpoints were not assessed. In addition, the observation period was limited to two weeks after the final administration. These aspects should be addressed in future studies focusing on efficacy, microbiome modulation, and longer-term outcomes. Nevertheless, our test probiotic preparation is intended for use in healthy animals to prevent and control postpartum uterine diseases, similar to a previous study in which intravaginal application of a *Lactobacillus* product reduced the incidence of metritis and overall uterine infections (Deng et al. 2015). In the case of the new medicinal products, additional experience gained during clinical efficacy tests will need to be considered to assess their safety. Additionally, the product’s safety should be re-evaluated once it is widely used in everyday clinical practice after approval (Elliot 2024).

## Conclusions

This target animal safety study demonstrated good local and systemic tolerability of the tested intravaginal probiotic formulation in healthy dairy cows, with no evidence of dose-dependent adverse effects. Observed time-related changes were transient and consistent with physiological variation rather than treatment-related toxicity. These findings establish a safety basis for further clinical development of the product and support subsequent studies evaluating efficacy and microbiome-related outcomes.

## Supplementary Information

Below is the link to the electronic supplementary material.

Supplementary Material 1

Supplementary Material 2

## Data Availability

The datasets generated during and/or analysed during the current study are available from the corresponding author on reasonable request.
