# Supplementary material for: Evaluation of the local tolerance and systemic safety of a novel intravaginal probiotic product in cows
Source: Vet Res Commun. 2026 May 11;50(4):316. doi: 10.1007/s11259-026-11264-7 (PMC13161294; doi:10.1007/s11259-026-11264-7)
Supplement: Supplementary file 1 — Supplementary Material 1 [file 11259_2026_11264_MOESM1_ESM.docx]

Table S2. Blood and urine parameters prior to the administration of the intravaginal probiotic preparation in cows.

| **Before** | | | | | | |
| --- | --- | --- | --- | --- | --- | --- |
| **Parameter** | **Unit** | **Reference Range** | **Control** | **1×** | **3×** | **5×** |
| **Aspartate Aminotransferase (AST)** | U/L | 78–132 | 96.5 (90.5–114.5) | 84.5 (70.5–112) | 87.5 (75.2–103.5) | 95 (87–152.5) |
| **Alanine Aminotransferase (ALT)** | U/L | 10–35 | 38.33 ± 4.46 | 33.00 ± 6.51 | 32.17 ± 9.77 | 35.17 ± 9.97 |
| **Alkaline Phosphatase (ALP)** | U/L | 20–150 | 158.33 ± 63.48 | 168.17 ± 64.00 | 200.83 ± 59.68 | 179.83 ± 41.83 |
| **Gamma-Glutamyl Transferase (GGT)** | U/L | 10–35 | 22.17 ± 6.79 | 26.50 ± 9.29 | 23.33 ± 9.42 | 28.50 ± 6.41 |
| **Total Protein** | g/L | 66–78 | 75.4 (73.7–76.9) | 79.6 (77.9–84.3) | 75.2 (73.6–76.4) | 73.9 (72.7–75.6) |
| **Albumin** | g/L | 25–38 | 33.1 (31.4–34) | 32.3 (31.3–33) | 32.5 (28.9–33.2) | 31.6 (30.6–32.4) |
| **Albumin/Globulin Ratio** |  | 0.8–1.2 | 0.8 (0.7–0.9) | 0.6 (0.5–0.7) | 0.8 (0.6–0.9) | 0.7 (0.7–0.8) |
| **Serum Amyloid A (quantitative)** | mg/L | < 20 | 13.2 (7.2–20.5) | 5.7 (5.3–6.6) | 11.1 (7.9–13.7) | 9.9 (7.9–11.8) |
| **Haptoglobin** | g/L | < 0.5 | 0.2 (0.2–0.2) | 0.1 (0.1–0.2) | 0.1 (0.1–0.2) | 0.2 (0.1–0.2) |
| **Glucose** | mmol/L | 2.2–5.5 | 4.3 (4.1–4.5) | 4.2 (4–4.5) | 4.3 (4.2–4.5) | 4.4 (3.9–4.6) |
| **Total Cholesterol** | mmol/L | 1.3–6.0 | 5.62 ± 1.49 | 4.47 ± 1.91 | 4.80 ± 1.15 | 4.67 ± 0.48 |
| **Triglycerides** | mmol/L | 0.08–0.20 | 0.06 ± 0.04 | 0.06 ± 0.02 | 0.07 ± 0.02 | 0.06 ± 0.02 |
| **Beta-Hydroxybutyrate (BHB)** | mmol/L | < 1.0 | 0.6 (0.5–0.7) | 0.7 (0.7–0.8) | 0.8 (0.7–0.9) | 0.7 (0.6–0.8) |
| **Non-Esterified Fatty Acids (NEFA)** | mmol/L | < 0.4 | 0.1 (0.1–0.1) | 0.1 (0.1–0.1) | 0.1 (0.1–0.1) | 0.1 (0.1–0.1) |
| **Urea** | mmol/L | 3–8 | 6.4 (6.1–6.7) | 6.7 (6.5–7) | 6.5 (6.3–6.8) | 6.6 (6.3–7.1) |
| **Creatinine** | µmol/L | 40–148 | 95 (88.5–98.5) | 90.5 (87.5–93.5) | 85.5 (83.5–92.8) | 89.5 (87.2–91.8) |
| **Phosphorus** | mmol/L | 1.47–2.63 | 1.7 (1.5–1.8) | 1.6 (1.6–1.7) | 1.7 (1.7–1.7) | 2 (1.8–2.2) |
| **Sodium** | mmol/L | 132–152 | 141.98 ± 0.71 | 142.07 ± 1.54 | 141.32 ± 0.83 | 142.17 ± 1.51 |
| **Potassium** | mmol/L | 3.9–5.8 | 5 (4.5–5.3) | 4.7 (4.6–4.8) | 4.6 (4.4–4.8) | 4.6 (4.5–4.7) |
| **Calcium** | mmol/L | 2.1–2.5 | 2.4 (2.3–2.4) | 2.3 (2.2–2.4) | 2.2 (2.2–2.3) | 2.2 (2.2–2.4) |
| **Magnesium** | mmol/L | 0.8–1.32 | 0.94 ± 0.04 | 0.95 ± 0.04 | 0.97 ± 0.15 | 0.99 ± 0.08 |
| **Chloride** | mmol/L | 95–110 | 103.17 ± 1.60 | 103.00 ± 2.19 | 104.00 ± 2.76 | 103.67 ± 1.97 |
| **Iron** | µmol/L | 20–35 | 24.93 ± 5.83 | 21.85 ± 5.02 | 26.33 ± 4.06 | 23.60 ± 6.23 |
| **Creatine Kinase (CK)** | U/L | 0–350 | 261.5 (179.2–481) | 223.5 (177.2–250.2) | 168 (166.5–207) | 202.5 (137.2–252) |
| **Lactate Dehydrogenase (LDH)** | U/L | 1740–2500 | 2163.5 (1870.8–2250.8) | 2126.5 (1925.8–2277) | 2124 (2003.8–2318.5) | 2138 (1222.8–2361) |
| **White blood cells (WBC)** | cells/L | 4.0 – 12.0 × 10⁹ | 9.88 ± 3.53 | 8.98 ± 2.26 | 10.15 ± 1.45 | 9.00 ± 2.14 |
| **Neutrophils** | cells/L | 0.6 – 4.0 × 10⁹ | 4.1 (2.6–6.4) | 3.9 (3.2–4) | 4.4 (3.7–4.6) | 3.5 (3.4–3.7) |
| **Lymphocytes** | cells/L | 2.0 – 7.0 × 10⁹ | 3.82 ± 0.89 | 3.90 ± 1.13 | 4.08 ± 1.47 | 4.18 ± 1.63 |
| **Monocytes** | cells/L | 0.025 – 0.84 × 10⁹ | 0.4 (0.4–0.6) | 0.4 (0.4–0.5) | 0.5 (0.4–0.5) | 0.4 (0.3–0.5) |
| **Eosinophils** | cells/L | 0 – 2.0 × 10⁹ | 0.6 (0.4–0.6) | 0.4 (0.3–0.5) | 0.4 (0.4–0.6) | 0.5 (0.3–1) |
| **Basophils** | cells/L | 0 – 0.2 × 10⁹ | 0.1 (0.1–0.1) | 0.1 (0.1–0.1) | 0.1 (0.1–0.1) | 0.1 (0.1–0.1) |
| **Red blood cells (RBC)** | cells/L | 5.0 – 10.0 × 10¹² | 6.27 ± 0.51 | 6.63 ± 0.51 | 6.75 ± 0.42 | 6.58 ± 0.53 |
| **Haemoglobin** | g/L | 80 – 150 | 104.83 ± 4.96 | 103.83 ± 5.98 | 109.83 ± 6.37 | 111.00 ± 9.34 |
| **Haematocrit** | % | 24 – 46 | 30.17 ± 1.17 | 29.50 ± 1.64 | 31.33 ± 1.63 | 31.50 ± 2.43 |
| **Platelets** | cells/L | 100 – 800 × 10⁹ | 309.17 ± 66.11 | 355.00 ± 133.91 | 280.00 ± 65.50 | 282.67 ± 128.85 |
| **Urine specific gravity** |  | 1.02 – 1.045 | 1 (1–1) | 1 (1–1) | 1 (1–1) | 1 (1–1) |
| **Urine pH** |  | 7.0-8.5 | 8.8 (8.1–9) | 8 (8–8.8) | 8.5 (8–9) | 8.2 (8–8.9) |

Normally distributed data are presented as mean±SD and non-normally distributed data as median and interquartile range (IQR) for each treatment group (Control, 1×, 3×, and 5×; n = 6/group). At this time point, cows were allocated to treatment groups but had not yet received any treatment. Parameters include haematology, biochemistry, and urinalysis.

Table S3. Blood and urine parameters one day after the 3rd administration of intravaginal probiotic preparation in cows.

| **After** | | | | | | |
| --- | --- | --- | --- | --- | --- | --- |
| **Parameter** | **Unit** | **Reference Range** | **Control** | **1×** | **3×** | **5×** |
| **Aspartate Aminotransferase (AST)** | U/L | 78–132 | 100.5 (92.5–112.2) | 94.5 (77.8–145.8) | 100.5 (85.5–116.2) | 106 (88.5–156.5) |
| **Alanine Aminotransferase (ALT)** | U/L | 10–35 | 43.33 ± 5.24 | 38.50 ± 6.66 | 37.50 ± 8.69 | 40.83 ± 11.57 |
| **Alkaline Phosphatase (ALP)** | U/L | 20–150 | 153.67 ± 55.60 | 161.50 ± 58.35 | 199.67 ± 61.94 | 187.83 ± 46.22 |
| **Gamma-Glutamyl Transferase (GGT)** | U/L | 10–35 | 26.33 ± 5.28 | 32.00 ± 8.76 | 26.67 ± 10.03 | 30.00 ± 7.18 |
| **Total Protein** | g/L | 66–78 | 74.2 (72.9–76.4) | 80.4 (75–84.4) | 72.8 (71.7–75.9) | 74.8 (74.6–76) |
| **Albumin** | g/L | 25–38 | 33.9 (33.2–35) | 33.1 (32.5–33.8) | 33.6 (31–34.3) | 32.5 (31.8–33.7) |
| **Albumin/Globulin Ratio** |  | 0.8–1.2 | 0.8 (0.7–0.9) | 0.7 (0.5–0.8) | 0.9 (0.7–0.9) | 0.7 (0.7–0.8) |
| **Serum Amyloid A (quantitative)** | mg/L | < 20 | 17 (7.5–30.8) | 7.6 (6.3–10) | 6.9 (4.7–9.2) | 9.8 (7.7–15.5) |
| **Haptoglobin** | g/L | < 0.5 | 0.2 (0.2–0.2) | 0.2 (0.1–0.3) | 0.2 (0.1–0.3) | 0.2 (0.1–0.3) |
| **Glucose** | mmol/L | 2.2–5.5 | 4.7 (4.5–4.8) | 4.7 (4.5–4.9) | 4.8 (4.8–4.9) | 4.6 (4.6–4.8) |
| **Total Cholesterol** | mmol/L | 1.3–6.0 | 5.75 ± 1.37 | 4.55 ± 1.76 | 4.80 ± 1.19 | 4.78 ± 0.45 |
| **Triglycerides** | mmol/L | 0.08–0.20 | 0.17 ± 0.05 | 0.15 ± 0.04 | 0.17 ± 0.02 | 0.15 ± 0.03 |
| **Beta-Hydroxybutyrate (BHB)** | mmol/L | < 1.0 | 0.3 (0.3–0.4) | 0.3 (0.3–0.4) | 0.3 (0.3–0.4) | 0.3 (0.3–0.4) |
| **Non-Esterified Fatty Acids (NEFA)** | mmol/L | < 0.4 | 0.4 (0.3–0.5) | 0.2 (0.2–0.3) | 0.2 (0.1–0.3) | 0.2 (0.1–0.2) |
| **Urea** | mmol/L | 3–8 | 3 (2.8–3.3) | 3 (2.8–3.2) | 3 (2.6–3.3) | 3.2 (2.9–3.3) |
| **Creatinine** | µmol/L | 40–148 | 87 (84.5–88.8) | 88 (84.8–89) | 78.5 (75–84.2) | 84.5 (81.2–88.5) |
| **Phosphorus** | mmol/L | 1.47–2.63 | 1.6 (1.6–1.7) | 1.6 (1.6–1.8) | 1.8 (1.7–1.8) | 1.8 (1.7–2) |
| **Sodium** | mmol/L | 132–152 | 140.87 ± 1.49 | 140.40 ± 1.12 | 140.32 ± 1.01 | 140.45 ± 1.42 |
| **Potassium** | mmol/L | 3.9–5.8 | 4.8 (4.8–5.1) | 4.8 (4.6–5) | 5 (4.8–5.2) | 4.9 (4.9–5) |
| **Calcium** | mmol/L | 2.1–2.5 | 2.3 (2.3–2.4) | 2.2 (2.2–2.4) | 2.3 (2.3–2.4) | 2.3 (2.2–2.4) |
| **Magnesium** | mmol/L | 0.8–1.32 | 1.01 ± 0.19 | 1.01 ± 0.12 | 0.95 ± 0.16 | 1.07 ± 0.14 |
| **Chloride** | mmol/L | 95–110 | 105.83 ± 1.72 | 105.00 ± 1.55 | 106.17 ± 0.98 | 105.33 ± 1.75 |
| **Iron** | µmol/L | 20–35 | 22.75 ± 2.20 | 21.80 ± 4.42 | 24.22 ± 4.73 | 23.92 ± 2.88 |
| **Creatine Kinase (CK)** | U/L | 0–350 | 237.5 (216.8–327.2) | 180.5 (155.8–205.2) | 176 (148.2–202.2) | 205.5 (146.8–254.5) |
| **Lactate Dehydrogenase (LDH)** | U/L | 1740–2500 | 2225 (2101.2–2412.5) | 2340 (2213.8–2380) | 2347.5 (2148.8–2546.2) | 2491.5 (2265.8–3003.8) |
| **White blood cells (WBC)** | cells/L | 4.0 – 12.0 × 10⁹ | 7.97 ± 2.69 | 7.10 ± 0.62 | 9.22 ± 1.06 | 8.83 ± 2.18 |
| **Neutrophils** | cells/L | 0.6 – 4.0 × 10⁹ | 3.5 (2–4.6) | 3.2 (2.8–3.3) | 4 (3.5–4.7) | 4 (3.1–5) |
| **Lymphocytes** | cells/L | 2.0 – 7.0 × 10⁹ | 3.27 ± 0.95 | 3.12 ± 0.63 | 3.70 ± 1.13 | 3.87 ± 1.36 |
| **Monocytes** | cells/L | 0.025 – 0.84 × 10⁹ | 0.4 (0.2–0.5) | 0.3 (0.2–0.5) | 0.3 (0.2–0.4) | 0.3 (0.3–0.4) |
| **Eosinophils** | cells/L | 0 – 2.0 × 10⁹ | 0.5 (0.5–0.6) | 0.4 (0.2–0.4) | 0.4 (0.4–0.7) | 0.3 (0.2–0.9) |
| **Basophils** | cells/L | 0 – 0.2 × 10⁹ | 0.1 (0.1–0.1) | 0.1 (0.1–0.1) | 0.1 (0.1–0.1) | 0.1 (0.1–0.1) |
| **Red blood cells (RBC)** | cells/L | 5.0 – 10.0 × 10¹² | 6.37 ± 0.49 | 7.07 ± 0.56 | 6.70 ± 0.36 | 6.87 ± 0.60 |
| **Haemoglobin** | g/L | 80 – 150 | 109.00 ± 7.87 | 111.50 ± 11.33 | 110.50 ± 8.69 | 117.67 ± 10.69 |
| **Haematocrit** | % | 24 – 46 | 30.50 ± 2.59 | 31.33 ± 2.58 | 31.33 ± 2.07 | 32.83 ± 2.64 |
| **Platelets** | cells/L | 100 – 800 × 10⁹ | 320.33 ± 77.37 | 359.50 ± 92.45 | 361.33 ± 95.20 | 299.00 ± 124.92 |
| **Urine specific gravity** |  | 1.02 – 1.045 | 1 (1–1) | 1 (1–1) | 1 (1–1) | 1 (1–1) |
| **Urine pH** |  | 7.0-8.5 | 9 (8.6–9) | 8.5 (8.5–8.5) | 8.5 (8.5–8.5) | 8.8 (8.5–9) |

Normally distributed data are presented as mean±SD and non-normally distributed data as median and interquartile range (IQR) for each treatment group (Control, 1×, 3×, and 5×; n = 6/group). Each cow received the treatment (see composition in Table 2) intravaginally in 10 ml volume for 3 consecutive days. Parameters include haematology, biochemistry, and urinalysis.

Table S4. Blood and urine parameters two weeks after the 3rd administration of intravaginal probiotic preparation in cows.

| **Two weeks after** | | | | | | |
| --- | --- | --- | --- | --- | --- | --- |
| **Parameter** | **Unit** | **Reference Range** | **Control** | **1×** | **3×** | **5×** |
| **Aspartate Aminotransferase (AST)** | U/L | 78–132 | 108.5 (79–129.8) | 89 (78.5–131) | 83.5 (78–107.8) | 107 (87.2–138) |
| **Alanine Aminotransferase (ALT)** | U/L | 10–35 | 41.33 ± 6.06 | 36.00 ± 5.97 | 35.83 ± 5.46 | 38.67 ± 7.47 |
| **Alkaline Phosphatase (ALP)** | U/L | 20–150 | 149.00 ± 66.05 | 157.83 ± 61.29 | 217.17 ± 68.46 | 174.83 ± 59.91 |
| **Gamma-Glutamyl Transferase (GGT)** | U/L | 10–35 | 21.33 ± 9.16 | 28.83 ± 9.33 | 25.83 ± 7.00 | 28.17 ± 4.88 |
| **Total Protein** | g/L | 66–78 | 75.8 (73–77.5) | 79.8 (74.6–82.1) | 72.4 (68.4–75.1) | 74.9 (71.9–78.3) |
| **Albumin** | g/L | 25–38 | 32.8 (32.3–34.3) | 31.7 (30.9–33) | 32.3 (30–33.1) | 31.4 (30.2–33.5) |
| **Albumin/Globulin Ratio** |  | 0.8–1.2 | 0.8 (0.7–0.8) | 0.6 (0.6–0.7) | 0.8 (0.7–0.9) | 0.7 (0.7–0.8) |
| **Serum Amyloid A (quantitative)** | mg/L | < 20 | 9.3 (5.6–17.9) | 9.1 (5–13.1) | 8.4 (7.3–9.4) | 9.2 (5.6–13.3) |
| **Haptoglobin** | g/L | < 0.5 | 0.4 (0.2–0.4) | 0.3 (0.2–0.3) | 0.3 (0.2–0.3) | 0.3 (0.3–0.4) |
| **Glucose** | mmol/L | 2.2–5.5 | 4.4 (4.4–4.6) | 4.6 (4.5–4.8) | 4.8 (4.7–5.3) | 4.4 (4.2–4.6) |
| **Total Cholesterol** | mmol/L | 1.3–6.0 | 5.27 ± 1.34 | 4.17 ± 1.72 | 4.42 ± 0.86 | 4.33 ± 0.33 |
| **Triglycerides** | mmol/L | 0.08–0.20 | 0.16 ± 0.03 | 0.14 ± 0.02 | 0.17 ± 0.04 | 0.14 ± 0.03 |
| **Beta-Hydroxybutyrate (BHB)** | mmol/L | < 1.0 | 0.5 (0.4–0.6) | 0.4 (0.3–0.4) | 0.4 (0.4–0.5) | 0.5 (0.4–0.6) |
| **Non-Esterified Fatty Acids (NEFA)** | mmol/L | < 0.4 | 0.2 (0.1–0.2) | 0.2 (0.2–0.2) | 0.2 (0.2–0.3) | 0.1 (0.1–0.1) |
| **Urea** | mmol/L | 3–8 | 3.9 (3.8–4.2) | 4 (3.7–4) | 3.8 (3.6–4.1) | 4.3 (4.1–4.4) |
| **Creatinine** | µmol/L | 40–148 | 97.5 (91.2–100.8) | 89.5 (88.2–90.8) | 87.5 (86–90.5) | 90.5 (90–93.2) |
| **Phosphorus** | mmol/L | 1.47–2.63 | 1.6 (1.5–1.8) | 1.5 (1.5–1.7) | 1.6 (1.5–1.6) | 1.7 (1.6–2) |
| **Sodium** | mmol/L | 132–152 | 141.75 ± 1.19 | 139.53 ± 2.10 | 139.32 ± 1.59 | 141.22 ± 1.81 |
| **Potassium** | mmol/L | 3.9–5.8 | 4.8 (4.6–5.1) | 4.8 (4.6–4.9) | 4.7 (4.5–4.7) | 4.7 (4.6–4.9) |
| **Calcium** | mmol/L | 2.1–2.5 | 2.3 (2.2–2.3) | 2.2 (2.2–2.3) | 2.2 (2.2–2.3) | 2.2 (2.1–2.3) |
| **Magnesium** | mmol/L | 0.8–1.32 | 1.06 ± 0.06 | 0.99 ± 0.07 | 1.03 ± 0.13 | 1.06 ± 0.08 |
| **Chloride** | mmol/L | 95–110 | 102.33 ± 2.16 | 102.33 ± 3.14 | 101.83 ± 1.17 | 103.17 ± 2.14 |
| **Iron** | µmol/L | 20–35 | 29.45 ± 2.35 | 28.18 ± 2.90 | 29.47 ± 4.95 | 25.88 ± 1.67 |
| **Creatine Kinase (CK)** | U/L | 0–350 | 168 (143–648.2) | 209.5 (192.5–744.8) | 168.5 (144.5–263) | 181 (164–183) |
| **Lactate Dehydrogenase (LDH)** | U/L | 1740–2500 | 2032.5 (1923–2511) | 2169.5 (2069.8–2372.8) | 2331.5 (2166.5–2568.5) | 2610.5 (2316.5–2990) |
| **White blood cells (WBC)** | cells/L | 4.0 – 12.0 × 10⁹ | 7.85 ± 2.24 | 7.13 ± 2.36 | 9.67 ± 1.92 | 8.52 ± 1.31 |
| **Neutrophils** | cells/L | 0.6 – 4.0 × 10⁹ | 3.2 (2.3–3.7) | 2.9 (2.6–3.2) | 3.7 (3.4–5.3) | 3.1 (2.9–3.3) |
| **Lymphocytes** | cells/L | 2.0 – 7.0 × 10⁹ | 3.50 ± 1.23 | 3.15 ± 1.43 | 3.90 ± 1.10 | 4.18 ± 1.20 |
| **Monocytes** | cells/L | 0.025 – 0.84 × 10⁹ | 0.4 (0.3–0.4) | 0.5 (0.3–0.5) | 0.5 (0.4–0.6) | 0.4 (0.4–0.5) |
| **Eosinophils** | cells/L | 0 – 2.0 × 10⁹ | 0.6 (0.4–0.7) | 0.2 (0.2–0.5) | 0.4 (0.3–1) | 0.5 (0.4–0.7) |
| **Basophils** | cells/L | 0 – 0.2 × 10⁹ | 0.1 (0.1–0.1) | 0.1 (0–0.1) | 0.1 (0.1–0.1) | 0.1 (0.1–0.1) |
| **Red blood cells (RBC)** | cells/L | 5.0 – 10.0 × 10¹² | 6.38 ± 0.58 | 6.73 ± 0.58 | 6.50 ± 0.33 | 6.72 ± 0.61 |
| **Haemoglobin** | g/L | 80 – 150 | 107.67 ± 4.89 | 107.00 ± 9.21 | 108.33 ± 8.29 | 114.67 ± 8.14 |
| **Haematocrit** | % | 24 – 46 | 30.33 ± 1.63 | 29.33 ± 2.50 | 29.83 ± 2.32 | 32.00 ± 1.67 |
| **Platelets** | cells/L | 100 – 800 × 10⁹ | 251.00 ± 105.03 | 312.83 ± 62.15 | 275.67 ± 94.60 | 271.00 ± 106.55 |
| **Urine specific gravity** |  | 1.02 – 1.045 | 1 (1–1) | 1 (1–1) | 1 (1–1) | 1 (1–1) |
| **Urine pH** |  | 7.0-8.5 | 9 (9–9) | 8.5 (8–9) | 8.5 (8–9) | 9 (9–9) |

Normally distributed data are presented as mean±SD and non-normally distributed data as median and interquartile range (IQR) for each treatment group (Control, 1×, 3×, and 5×; n = 6/group). Each cow received the treatment (see composition in Table 2) intravaginally in 10 ml volume for 3 consecutive days. Parameters include haematology, biochemistry, and urinalysis.
