# Supplementary material for: Evaluation of the local tolerance and systemic safety of a novel intravaginal probiotic product in cows
Source: Vet Res Commun. 2026 May 11;50(4):316. doi: 10.1007/s11259-026-11264-7 (PMC13161294; doi:10.1007/s11259-026-11264-7)
Supplement: Supplementary file 2 — Supplementary Material 2 [file 11259_2026_11264_MOESM2_ESM.docx]

Table S1. Analytical methods used for serum biochemical and inflammatory parameters

| **Parameter** | **Analytical method/Reagent** | **Manufacturer** |
| --- | --- | --- |
| Aspartate Aminotransferase (AST) | Kinetic optimised UV test | CliniChem Kft. |
| Alanine Aminotransferase (ALT) | Kinetic optimised UV test | CliniChem Kft. |
| Alkaline Phosphatase (ALP) | Optimised DGKC method | CliniChem Kft. |
| Gamma-Glutamyl Transferase (GGT) | Modified Szasz kinetic colorimetric method | CliniChem Kft. |
| Total bilirubin | Dichloroaniline (DCA), photometric test | DiaSys Diagnostic Systems GmbH |
| Direct bilirubin | Dichloroaniline (DCA), photometric test | DiaSys Diagnostic Systems GmbH |
| Total protein | Biuret method | CliniChem Kft. |
| Albumin | Bromocresol green colorimetric method | CliniChem Kft. |
| Glucose | Enzymatic colorimetric (GOD/POD/PAP) | CliniChem Kft. |
| Total cholesterol | Enzymatic colorimetric (PAP) | CliniChem Kft. |
| Triglycerides | Enzymatic colorimetric (PAP) | CliniChem Kft. |
| Beta-hydroxybutyrate (BHB) | Enzymatic (BHB dehydrogenase) | DiaSys Diagnostic Systems GmbH |
| Non-Esterified Fatty Acids (NEFA) | Enzymatic (acyl-CoA synthetase, ACS) | DiaSys Diagnostic Systems GmbH |
| Urea | Enzymatic optimised UV test | CliniChem Kft. |
| Creatinine | Jaffé method | CliniChem Kft. |
| Inorganic phosphorus | Phosphomolybdate UV method | CliniChem Kft. |
| Calcium | Arsenazo method | CliniChem Kft. |
| Magnesium | Xylidyl blue method | CliniChem Kft. |
| Iron | Ferene photometric test | CliniChem Kft. |
| Sodium | Ion-selective electrode | Diagnosticum Zrt. |
| Potassium | Ion-selective electrode | Diagnosticum Zrt. |
| Creatine kinase (CK) | CK-NAC test | CliniChem Kft. |
| Lactate dehydrogenase (LDH) | DGKC-recommended UV test | CliniChem Kft. |
| Serum amyloid A (SAA) | Turbidimetric assay, Vet-SAA ’Eiken’ | Eiken Chemical Co. LTD |
| Haptoglobin | Bovine Hp test kit | Tridelta Development Ltd. |

All serum biochemical and inflammatory parameters were analysed using an automated clinical chemistry analyser (Advia 1800; Siemens) according to the manufacturers’ instructions.
